# Performance of seven serological assays for diagnosing tularemia

**DOI:** 10.1186/1471-2334-14-234

**Published:** 2014-05-05

**Authors:** Valérie Chaignat, Marina Djordjevic-Spasic, Anke Ruettger, Peter Otto, Diana Klimpel, Wolfgang Müller, Konrad Sachse, George Araj, Roland Diller, Herbert Tomaso

**Affiliations:** 1 Institute of Bacterial Infections and Zoonoses, Friedrich-Loeffler-Institut (Federal Research Institute for Animal Health), Naumburger Str. 96a, Jena 07743, Germany; 2 Clinical Centre Niš, Clinic for Infectious Diseases, Bulevar Zorana Djindjica 48, Niš 18000, Serbia; 3 Institute of Molecular Pathogenesis, Friedrich-Loeffler-Institut (Federal Research Institute for Animal Health), Naumburger Str. 96a, Jena 07743, Germany; 4 Clinical Microbiology, Department of Pathology & Laboratory Medicine, American University of Beirut Medical Center, Beirut 1107-2020, Lebanon

**Keywords:** Serology, Tularemia, Diagnostic sensitivity, Diagnostic specificity

## Abstract

**Background:**

Tularemia is a rare zoonotic disease caused by the Gram-negative bacterium *Francisella tularensis*. Serology is frequently the preferred diagnostic approach, because the pathogen is highly infectious and difficult to cultivate. The aim of this retrospective study was to determine the diagnostic accuracy of tularemia specific tests.

**Methods:**

The *Serazym*®Anti-*Francisella tularensis* ELISA, Serion ELISA *classic Francisella tularensis* IgG/IgM, an in-house ELISA, the VIRapid® Tularemia immunochromatographic test, an in-house antigen microarray, and a Western Blot (WB) assay were evaluated. The diagnosis tularemia was established using a standard micro-agglutination assay. In total, 135 sera from a series of 110 consecutive tularemia patients were tested.

**Results:**

The diagnostic sensitivity and diagnostic specificity of the tests were VIRapid (97.0% and 84.0%), Serion IgG (96.3% and 96.8%), Serion IgM (94.8% and 96.8%), Serazym (97.0% and 91.5%), in-house ELISA (95.6% and 76.6%), WB (93.3% and 83.0%), microarray (91.1% and 97.9%).

**Conclusions:**

The diagnostic value of the commercial assays was proven, because the diagnostic accuracy was >90%. The diagnostic sensitivity of the in-house ELISA and the WB were acceptable, but the diagnostic accuracy was <90%. Interestingly, the antigen microarray test was very specific and had a very good positive predictive value.

## Background

Tularemia is a zoonotic disease caused by *Francisella tularensis*, a highly infectious, facultative intracellular, Gram-negative bacterium. The clinical presentation in humans depends on the route of infection and varies from relatively mild skin lesions and lymphadenopathy to life-threatening pneumonia and/or septicemia [1]. Due to the low infectious dose and the ease of dissemination by aerosols, this bacterium was categorized as a highly dangerous biological agent by the Centers for Disease Control in Atlanta (Georgia, USA) [2-4].

The diagnostic repertoire comprises cultivation methods, PCR assays, and serological tests [5-8]. Because of the high risk of laboratory infections associated with cultivation of *F. tularensis*, serology is frequently preferred. Serological assays are usually easy to perform, allow high throughput, and cause little risk of infection for laboratory workers [9-12]. However, some patients never seroconvert, while others stay seropositive for years after an infection, and/or may have cross- reacting antibodies due to infections with other bacteria (e.g. *Yersinia* spp.*, Brucella* spp.) [13,14].

The aim of this study was to compare novel assays with well-established diagnostic tools using a comprehensive collection of human sera.

## Methods

### Serum samples

The present retrospective study was conducted in accordance with the STARD guidelines to assess the diagnostic accuracy and the clinical value of the respective assays [15,16]. The study population included a consecutive series of 110 patients in an endemic area of tularemia in Serbia that had a history of potential risk of exposure and/or had clinical symptoms compatible with tularemia. In accordance with WHO guidelines, patients with typical symptoms were regarded as tularemia cases, if at least one serum sample was positive in the micro-agglutination assay (MAT) for tularemia [9]. If the first serum sample was negative, paired serum samples were tested after two weeks or later. In total 135 sera were collected between 1999 and 2009. Patients were excluded, if documentation was not complete or if the sample volume was not sufficient to perform all diagnostic tests assessed in this study. All patients signed an informed consent and the use of the samples for this study was granted by the Ethics Committee of the Medical Faculty, Niš University (Number 01-4608-3, 9^th^ July 2009).

In order to assess potential cross-reactivity, 94 sera of humans without known history of tularemia were tested including 20 seropositive samples from patients with culture-proven brucellosis from Lebanon. The company “Mikrogen” (Martinsried, Germany) kindly provided 74 sera collected from blood donors in Germany, which had been purchased from the Bavarian Red Cross (Munich, Germany). All control sera were anonymized in relation to patient data.

### Assays

#### MAT assay

This test was performed in Serbia to establish the diagnosis and was regarded as the reference method in this study. Briefly, serial 2-fold dilutions of sera (25 μl) were mixed with an equal volume of formalin-inactivated *F. tularensis* subsp. *holarctica* (LVS) whole cell suspension (OD_560_ = 1.0). The reactions were performed in round-bottom microtiter-plates (96-well; NUNC, Roskilde, Denmark). The plates were read out after incubation at 37°C for 18 h. Agglutinations at dilutions of 1:20 or higher were considered MAT positive.

**Figure 1:**
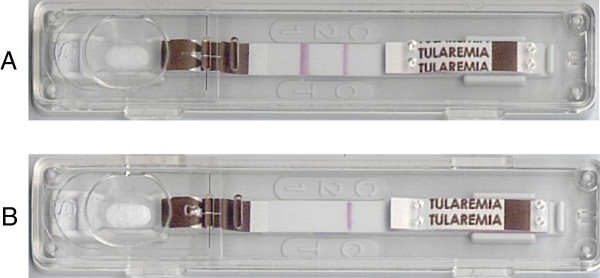
**Hand-held immunochromatographic test for the serological diagnosis of tularemia (VIRapid®; Vircell S.L., Santa Fé, Spain).** A test is interpreted as positive, if the specific line and the control line are positive **(A)**. The test is valid, but negative, if only the control line is visible **(B)**.

All commercial assays were performed and interpreted according to the manufacturer’s instructions:

The *Serazym*® Anti-*Francisella tularensis* ELISA **(**Serazym ELISA**) (**Seramun Diagnostica GmbH, Heidesee OT Wolzig, Germany**)** detects all classes of antibodies. The Serion ELISA *classic Francisella tularensis* IgG/IgM (IgG Serion ELISA/IgM Serion ELISA) **(**Institut Virion/Serion GmbH, Würzburg, Germany) allows separate detection of IgG and IgM. The VIRapid® (VIRapid) (Vircell S.L., Santa Fé, Spain) is an immunochromatographic lateral flow test (ICT) (Figure 1).

The *Yersinia recom*line kit (*Yersinia* IgG 2.0 kit, Mikrogen) was used to determine, which sera had anti-*Yersinia* antibodies that could potentially cause cross-reactions.

#### In-house developed assays

The in-house ELISA was designed to detect anti-*Francisella* IgG antibodies. The assay published by Porsch-Ozcürümez et al. [11] was performed with some modifications. Briefly, the coating antigen was a purified lipopolysaccharide (LPS) obtained from *F. tularensis* subsp. *holarctica* (ATCC 29684) (Micromun, Greifswald, Germany). The secondary antibody was a horseradish peroxidase-conjugated goat anti-human IgG (Millipore, Schwalbach, Germany). Receiver operating characteristic (ROC) curves were used to determine the cut-off value (MedCalc Software Version 13.0.4, Oostend, Belgium).

The Western Blot assay (WB) was developed as a modification of the test published by Schmitt et al. [10]. The *F. tularensis* LPS was purchased from Micromun. The secondary antibody was a purified recombinant protein A/G, which was alkaline phosphatase labeled (Pierce Biotechnology, Rockford, Illinois, USA) diluted 1:5,000**.**

The microarray detecting anti-*Francisella* antibodies used two different preparations of antigen: Whole-cell bacterial antigen of *Francisella tularensis* subsp. *holarctica* strain LVS and commercially available purified LPS were compared. Whole-cell bacterial antigen was obtained after growth for 48 h on Cysteine Heart Agar enriched with chocolatized red blood cells at 37°C in ambient atmosphere. Bacterial pellets were harvested and diluted in phosphate-buffered saline containing 5–20 mM sucrose to final antigen concentrations between 0.5 and 1 mg protein/ml. Subsequently, the antigen preparations were spotted onto microarray chips as previously described [17] and assembled in the ArrayStrip 8-well microtiter strip format (Alere Technologies GmbH, Jena, Germany). To evaluate the impact of the secondary antibody (Protein A/G-HRP, Thermo Fisher Scientific GmbH, Schwerte, Germany) on the background, it was added to the tularemia antigens without the patient sera. Results from this analysis showed no direct reactivity due to the secondary antibody.

#### Antigen-antibody reaction in array strip vessels

Examination of sera was conducted using the Protein Binding Kit (Alere Technologies GmbH) with the following modifications: The incubation buffer P1 was supplemented with 1% (w/v) milk powder (Marvel; Premier International Foods UK Ltd, Dublin, Ireland), and blocking buffer consisted of P1 containing 3% (w/v) milk powder. All incubation and washing steps were performed at 37°C using a heated horizontal tube shaker (BioShake iQ; Quantifoil Instruments GmbH, Jena, Germany). The ArrayStrips were initially conditioned with 100 μl of incubation buffer at 400 strokes per minute (spm) for 5 min. The supernatant was discarded and the microarrays were blocked by incubation with 100 μl blocking buffer at 300 spm for 5 min. Prior to incubation, serum samples were diluted 1:100 in incubation buffer in a separate tube. After transfer of 100 μl of diluted serum into the array vessel, the antigen-antibody reaction was allowed to proceed at 300 spm for 30 min. The supernatant was discarded, and the arrays were washed using 150 μl incubation buffer at 400 rpm for 5 min. The supernatant was again discarded and the arrays were incubated with 100 μl of a 1:10,000 dilution of HRP-conjugated Protein A/G (Thermo Fisher Scientific GmbH) in incubation buffer at 300 spm for 30 min. Subsequently, the ArrayStrips were washed in two steps as above. Finally, 100 μl of the ready-to-use HRP substrate D1 (part of the Protein Binding Kit, Alere Technologies GmbH) were added for staining (25°C, 10 min, no shaking). Before the final reading, the staining solution was completely removed. The outcome of the antigen-antibody reaction was measured using an ArrayMate transmission reader (Alere Technologies GmbH). Signal intensity data were processed using the Iconoclust software, version 3.3 (Alere Technologies GmbH). Pilot tests with positive and negative control sera were used to empirically determine a cut-off value of 0.3 intensity units (data not shown).

The diagnostic value was determined based on the following parameters: Diagnostic sensitivity = [TP/(TP + FN)] × 100; Diagnostic specificity = [TN/(TN + FP)] × 100; Positive Predictive Value (PPV) = [TP/(TP + FP)] × 100; Negative Predictive Value (NPV) = [TN/(TN + FN)] × 100; Diagnostic accuracy = (TP + TN)/(TP + TN + FP + FN) × 100. (TP = true positive, TN = true negative, FN = false negative, FP = false positive).

## Results

**Figure 2:**
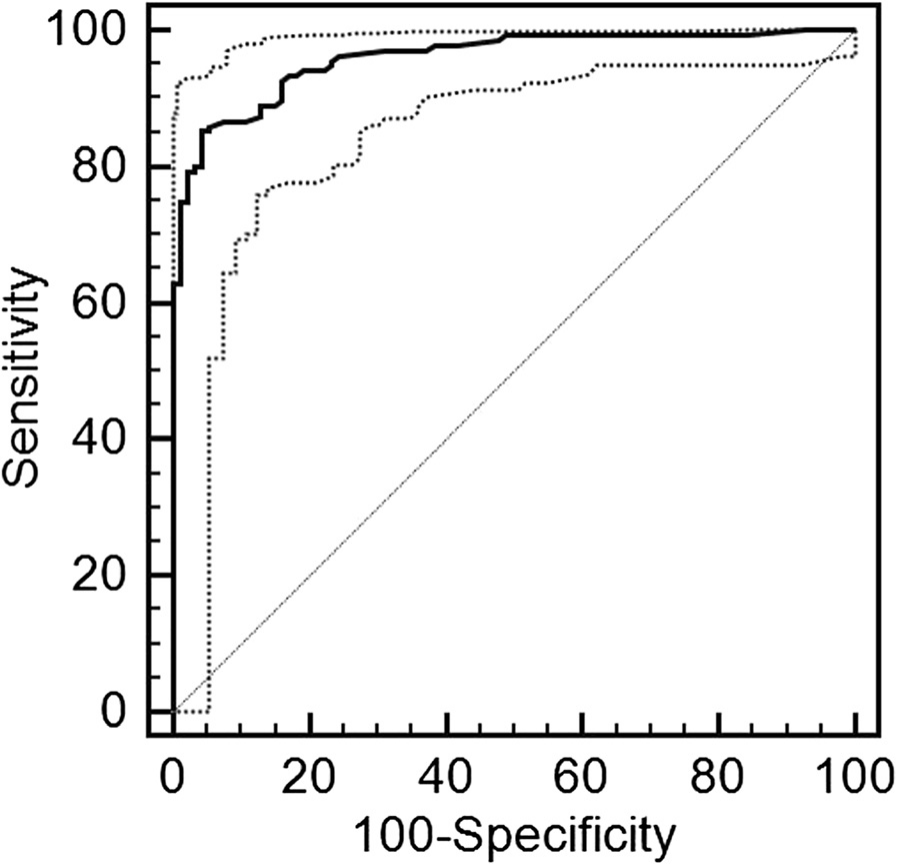
Receiver operating characteristic (ROC) curves were used to determine the cut-off value for the in-house ELISA (MedCalc software version 13.0.4, Oostend, Belgium).

The parameters used to assess the diagnostic accuracy for all serological assays are presented in Table 1. The diagnostic sensitivity and diagnostic specificity of the tests were: VIRapid (97.0% and 84.0%), Serion IgG (96.3% and 96.8%), Serion IgM (94.8% and 96.8%), Serazym (97.0% and 91.5%), in-house ELISA (cut-off = 0.18; 95.6% and 76.6%), WB (93.3% and 83.0%), microarray (91.1% and 97.9%). The cut-off for the in-house ELISA was set to 0.18 based on a ROC analysis in order to achieve a relatively high diagnostic sensitivity (Figure 2, Additional file 1).

**Table 1:** Performance of serological assays for diagnosing tularemia

	**In-house ELISA**	**In-house ELISA**	**VIRapid**	**WB**	**Serazym ELISA**	**Serion ELISA**	**Serion ELISA**	**Array**
	**Cut-off = 0.18**	**Cut-off = 0.33**				**IgG**	**IgM**	
True positive	129	118	131	126	131	130	128	123
False negative	6	17	4	9	4	5	7	12
False positive	22	12	15	16	8	3	3	2
True negative	72	82	79	78	86	91	91	92
Diagnostic sensitivity [%]	95.6	87.4	97.0	93.3	97.0	96.3	94.8	91.1
Diagnostic specificity [%]	76.6	87.2	84.0	83.0	91.5	96.8	96.8	97.9
PPV [%]	85.4	90.8	89.7	88.7	94.2	97.7	97.7	98.4
NPV [%]	92.3	82.8	95.2	89.7	95.6	94.8	92.9	88.5
Diagnostic accuracy [%]	87.8	87.3	91.7	89.1	94.8	96.5	95.6	93.9

The following tests were evaluated: Western blot (WB); Serion ELISA *classic* Francisella tularensis IgG/IgM (Institut Virion\Serion GmbH, Würzburg, Germany) (IgG and IgM Serion ELISA); *Serazym*® Anti-Francisella tularensis (Seramun Diagnostica GmbH, Heidesee OT Wolzig, Germany) (Serazym ELISA); VIRapid® Tularemia (Vircell S.L., Santa Fé, Spain) (VIRapid); antigen microarray (array) and an in-house ELISA. (TP = true positive, TN = true negative, FN = false negative, FP = false positive).

The diagnostic value was determined based on the following parameters: Diagnostic sensitivity = [TP/(TP + FN)] × 100; Diagnostic specificity = [TN/(TN + FP)] × 100; Positive Predictive Value (PPV) = [TP/(TP + FP)] × 100; Negative Predictive Value (NPV) = [TN/(TN + FN)] × 100; Diagnostic accuracy = (TP + TN)/(TP + TN + FP + FN)×100.

The microarray test with spotted whole antigen of *F. tularensis* was used for the final evaluation, because pilot experiments showed that spotted LPS lead to many false negative results. The results of the *Yersinia recom*line kit showed that 48% of the sera of tularemia cases (65/135) and 39% of non-tularemia sera (37/94) had antibodies against *Yersinia* spp., but their relevance regarding cross-reactions could not be determined. In the microarray no cross-reactions with anti-*Brucella* antibodies in 20 serologically positive sera of culture-proven brucellosis patients were observed in the microarray. Cross-reactions with anti-*Brucella* antibodies were observed in the following assays: VIRapid [7], Serion IgG [2], Serion IgM [3], Serazym [2], in-house ELISA [10], WB [10].

## Discussion

Tularemia is a zoonotic disease that has broken out naturally in many countries in recent years. The disease requires special attention because *F. tularensis* can be used as a bioterrorist agent [3,4,18]. Infections may remain undiagnosed, because the symptoms of tularemia are frequently non-specific [17,18]. This has serious consequences, because *F. tularensis* is resistant to cephalosporins and delayed adequate antibiotic therapy can lead to complications or even death [19-21]. In the past, several serological assays have been established and evaluated [6]. However, laboratories still face a capacity gap regarding reliable bedside tests and simple tools to assess cross-reactions. Although tularemia is endemic in many European countries, cases occur only sporadically [18]. Therefore, laboratories are requested to perform diagnostic tests for tularemia only rarely, which may make it less attractive to set up and validate ELISAs and WB assays. For field-use, a non-commercial in-house ICT was developed to support a presumptive diagnosis of tularemia [5]. However, routine laboratories and other first responders need ready-to-use commercial products that are certified for *in vitro* diagnostic use.

In this study, seven different serological assays for tularemia were evaluated including a commercially available ICT, and an antigen microarray. The assays were tested with a comprehensive number of sera obtained from tularemia cases and a collection of potentially cross-reacting sera with anti-*Brucella* and anti-*Yersinia* antibodies. The commercial ELISA assays showed excellent performance regarding diagnostic specificity and diagnostic sensitivity and proved their diagnostic value.

Schmitt et al. [10] showed that the combination of an in-house ELISA and a WB assay was suitable for screening and found no cross-reactions with antibodies against *Brucella* spp., *E. coli*, *Salmonella* spp., or *Yersinia enterocolitica*. Although the in-house ELISA in our study had an acceptable diagnostic sensitivity of 95.6% (cut-off = 0.18), the diagnostic accuracy of this ELISA and also of the WB assays established in this study were not as expected, because the correspondence with the reference test was <90%. This might be explained by the use of different staining protocols to detect the antigen-antibody reaction. In other studies, anti-human anti-IgG antibodies were used as secondary antibodies, whereas we used alkaline phosphatase-labeled protein A/G for the WB assay and protein G labeled with HRP for the in-house ELISA. This combination was chosen, because it allows the use of the assays for samples of various mammal species, which is important for screening the animal reservoir and indicator animals [6,10,11]. Interestingly, a recently published competitive ELISA using biotin-labelled anti-LPs Mabs, streptavidin-peroxidase and tetramethylbenzidine as enzyme substrate allowed screening of humans (and animals, i.e. rabbits and mice) with a diagnostic sensitivity and diagnostic specificity of 93.9 and 96.1%, respectively [22].

The VIRapid assay has a very good diagnostic sensitivity (97.0%) and is a useful hand-held test kit for field or bedside use, when only small numbers of samples need to be tested. This is in line with the good results obtained in a study in Turkey that included 236 sera of 109 tularemia cases where the test showed a diagnostic sensitivity of 99.3% and a diagnostic specificity of 94.6%. A study in Spain with 321 patients also found a diagnostic sensitivity of 95.5% and a diagnostic specificity of 100% [23]. In Turkey, 4 out of 50 (8%) sera of brucellosis patients cross-reacted, while in the present study, this was observed in 7 out of 20 serologically and culture-proven *Brucella* positive samples [24].

The microarray showed a good diagnostic specificity (97.9%) and may be a promising tool for simultaneous detection of antibodies to different antigens and for assessing cross-reactions. Therefore, studies are ongoing to combine *Francisella* antigens with those of other zoonotic pathogens on a microarray. Anti-*Yersinia* antibodies can be expected in approximately one third of the population and cross-reactions with tularemia might play a role [25]. However, our data do not allow reliable conclusions.

A limitation of this study is the relatively low number of non-tularemia sera (n = 94). Therefore, the value of these tests could not be assessed for use in large-scale seroprevalence studies.

## Conclusion

In our hands, the commercial ELISA assays have proven to be the method of choice for testing large numbers of samples. The VIRapid is of practical use as a bedside diagnostic assay to corroborate a clinical diagnosis, but it is neither designed nor useful for high throughput screening. The microarray is a promising tool for assessing cross-reactions and studies are ongoing to combine these targets with other zoonotic pathogens.

## Competing interests

The VIRapid Tularemia assay was kindly provided by Vircell (Vircell S.L., Santa Fé, Spain).

## Authors’ contributions

VC supervised the ELISA, WB and hand-held ICT and drafted the manuscript. MD investigated and treated the patients in Serbia. AR conducted all experiments using the protein microarray, processed the numerical data and participated in the design of the serology microarray. PO supervised ELISAs and WB and drafted the manuscript. DK and WM established the in-house ELISA and the WB. KS conceived the design of the serology microarray, participated in data processing and wrote part of the manuscript. GA diagnosed the brucellosis cases in Lebanon and revised the manuscript. RD performed the ROC analysis and drafted parts of the manuscript. HT designed the study, coordinated the experiments, and finalized the manuscript. All authors read and approved the final manuscript.

## Pre-publication history

The pre-publication history for this paper can be accessed here:

http://www.biomedcentral.com/1471-2334/14/234/prepub

## Supplementary Material

Additional file 1 Receiver operating characteristic (ROC) curves data used to determine the cut-off value for the in-house ELISA (MedCalc Software Version 13.0.4, Oostend, Belgium). Click here for file
