# Supplementary material for: Performance of seven serological assays for diagnosing tularemia
Source: BMC Infect Dis. 2014 May 5;14:234. doi: 10.1186/1471-2334-14-234 (PMC4021340; doi:10.1186/1471-2334-14-234)
Supplement: Additional file 1 — Receiver operating characteristic (ROC) curves data used to determine the cut-off value for the in-house ELISA (MedCalc Software Version 13.0.4, Oostend, Belgium). [file 1471-2334-14-234-S1.pdf]

ROC curve

Variable	Test
Classification variable	Diagnose

Sample size		229
Positive group :	Diagnose = 1	135
Negative group :	Diagnose = 0	94

Disease prevalence (%)	unknown

Area under the ROC curve (AUC)

Area under the ROC curve (AUC)	0,960
Standard Error[a]	0,0115
95% Confidence interval[b]	0,937 to 0,982
z statistic	40,046
Significance level P (Area=0.5)	<0,0001

[a] DeLong et al., 1988
[b] AUC ± 1.96 SE

Youden index

Youden index J	0,8093
Associated criterion	>0,42

Criterion values and coordinates of the ROC curve [Hide]

Criterion	Sensitivity	95% CI	Specificity	95% CI	+LR	-LR
≥0,03	100,00	97,3 - 100,0	0,00	0,0 - 3,8	1,00	
>0,05	100,00	97,3 - 100,0	7,45	3,0 - 14,7	1,08	0,00
>0,06	99,26	95,9 - 100,0	15,96	9,2 - 25,0	1,18	0,046
>0,1	99,26	95,9 - 100,0	51,06	40,5 - 61,5	2,03	0,015
>0,11	98,52	94,8 - 99,8	52,13	41,6 - 62,5	2,06	0,028
>0,12	97,78	93,6 - 99,5	58,51	47,9 - 68,6	2,36	0,038
>0,13	97,78	93,6 - 99,5	61,70	51,1 - 71,5	2,55	0,036
>0,14	97,04	92,6 - 99,2	62,77	52,2 - 72,5	2,61	0,047
>0,16	97,04	92,6 - 99,2	69,15	58,8 - 78,3	3,15	0,043
>0,17	96,30	91,6 - 98,8	75,53	65,6 - 83,8	3,94	0,049
>0,18	95,56	90,6 - 98,4	76,60	66,7 - 84,7	4,08	0,058
>0,19	94,81	89,6 - 97,9	76,60	66,7 - 84,7	4,05	0,068
>0,2	94,07	88,7 - 97,4	77,66	67,9 - 85,6	4,21	0,076
>0,23	94,07	88,7 - 97,4	80,85	71,4 - 88,2	4,91	0,073
>0,24	93,33	87,7 - 96,9	81,91	72,6 - 89,1	5,16	0,081
>0,25	93,33	87,7 - 96,9	82,98	73,8 - 89,9	5,48	0,080
>0,26	92,59	86,8 - 96,4	84,04	75,0 - 90,8	5,80	0,088
>0,27	89,63	83,2 - 94,2	84,04	75,0 - 90,8	5,62	0,12
>0,28	88,89	82,3 - 93,6	85,11	76,3 - 91,6	5,97	0,13
>0,31	88,89	82,3 - 93,6	87,23	78,8 - 93,2	6,96	0,13
>0,33	87,41	80,6 - 92,5	87,23	78,8 - 93,2	6,85	0,14
>0,34	86,67	79,7 - 91,9	89,36	81,3 - 94,8	8,15	0,15
>0,37	86,67	79,7 - 91,9	92,55	85,3 - 97,0	11,64	0,14
>0,38	85,93	78,9 - 91,3	94,68	88,0 - 98,3	16,15	0,15
>0,4	85,19	78,1 - 90,7	94,68	88,0 - 98,3	16,01	0,16
>0,42	85,19	78,1 - 90,7	95,74	89,5 - 98,8	20,02	0,15
>0,66	80,00	72,3 - 86,4	95,74	89,5 - 98,8	18,80	0,21
>0,68	80,00	72,3 - 86,4	96,81	91,0 - 99,3	25,07	0,21
>0,7	79,26	71,4 - 85,8	96,81	91,0 - 99,3	24,83	0,21

>0,72	79,26	71,4 - 85,8	97,87	92,5 - 99,7	37,25	0,21
>0,94	74,81	66,6 - 81,9	97,87	92,5 - 99,7	35,16	0,26
>1	74,81	66,6 - 81,9	98,94	94,2 - 100,0	70,33	0,25
>1,5	62,96	54,2 - 71,1	98,94	94,2 - 100,0	59,19	0,37
>1,6	62,96	54,2 - 71,1	100,00	96,2 - 100,0		0,37
>3,37	0,00	0,0 - 2,7	100,00	96,2 - 100,0		1,00
